# Supplementary material for: Chemotaxonomic Identification of Key Taste and Nutritional Components in ‘Shushanggan Apricot’ Fruits by Widely Targeted Metabolomics
Source: Molecules. 2022 Jun 16;27(12):3870. doi: 10.3390/molecules27123870 (PMC9227342; doi:10.3390/molecules27123870)
Supplement: Supplementary file 1 [file molecules-27-03870-s001.zip › Table S4.pdf]

Table S4. The test ranges, regression equations, correlation coefficients, and retention time of soluble sugars (sucrose, glucose and fructose) and organic acids (malic acid and citric acid) peaks

Compound name	Test range (mg/mL)	Regression equation	Coefficient R^2	Retention time
Sucrose	0.50-5.00	Y = 2.20e+006 X + 6.52e+005	0.998848	12.783
Glucose	0.50-5.00	Y = 1.84e+006 X - 8.11e+005	0.998732	9.197
Fructose	0.50-5.00	Y = 1.83e+006 X – 9.29e+005	0.997089	11.581
Malic acid	0.03215-0.50000	Y = 9.56e+005 X + 3.78e+003	0.998801	5.752
Citric acid	0.03215-0.50000	Y = 1.15e+006 X + 2.43e+004	0.999416	9.847
