# Supplementary figures and images for: Chemotaxonomic Identification of Key Taste and Nutritional Components in ‘Shushanggan Apricot’ Fruits by Widely Targeted Metabolomics
Source: Molecules. 2022 Jun 16;27(12):3870. doi: 10.3390/molecules27123870 (PMC9227342; doi:10.3390/molecules27123870)

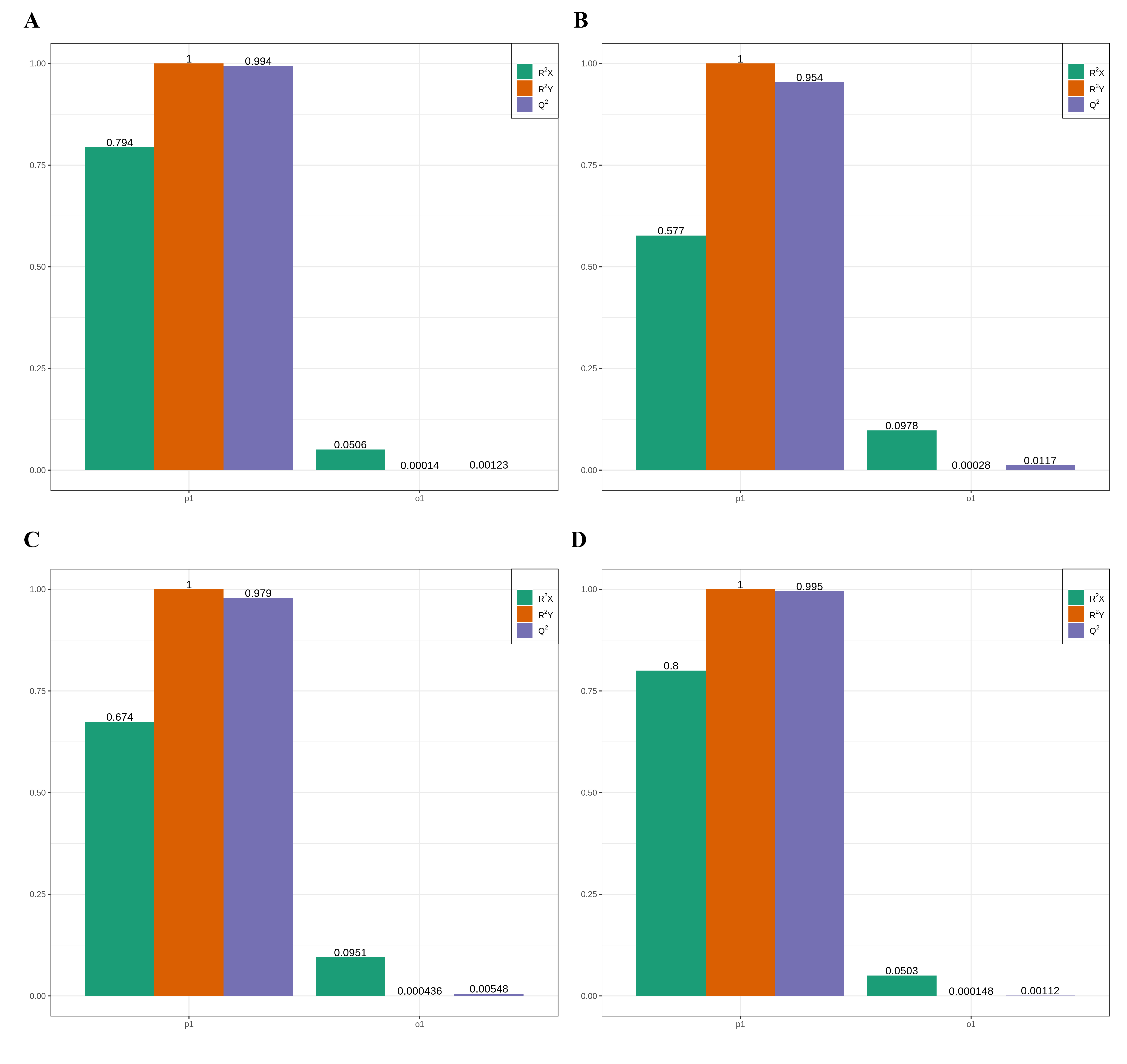

Supplement: Supplementary file 1 [file molecules-27-03870-s001.zip › Figure S1.jpg]

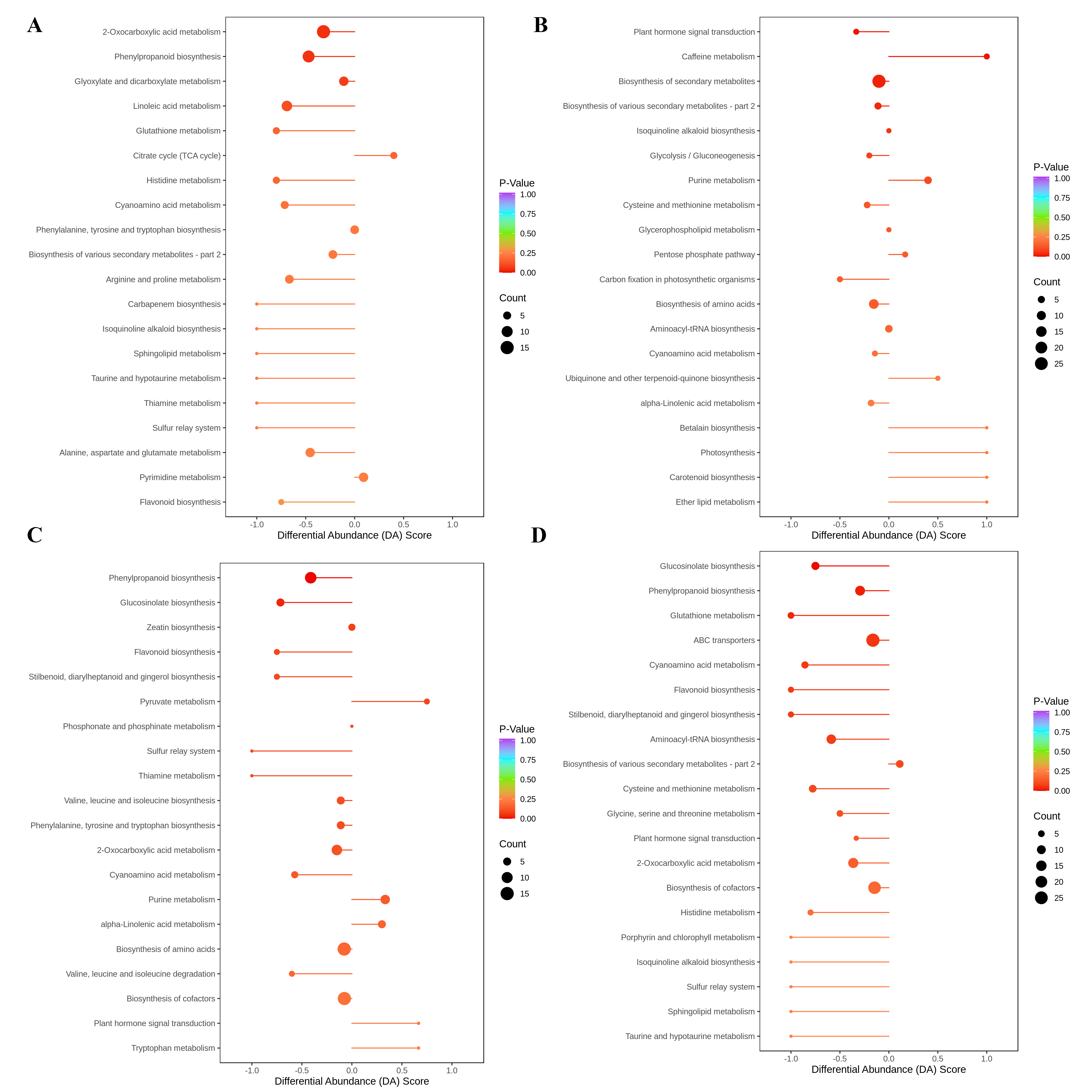

Supplement: Supplementary file 1 [file molecules-27-03870-s001.zip › Figure S2.jpg]

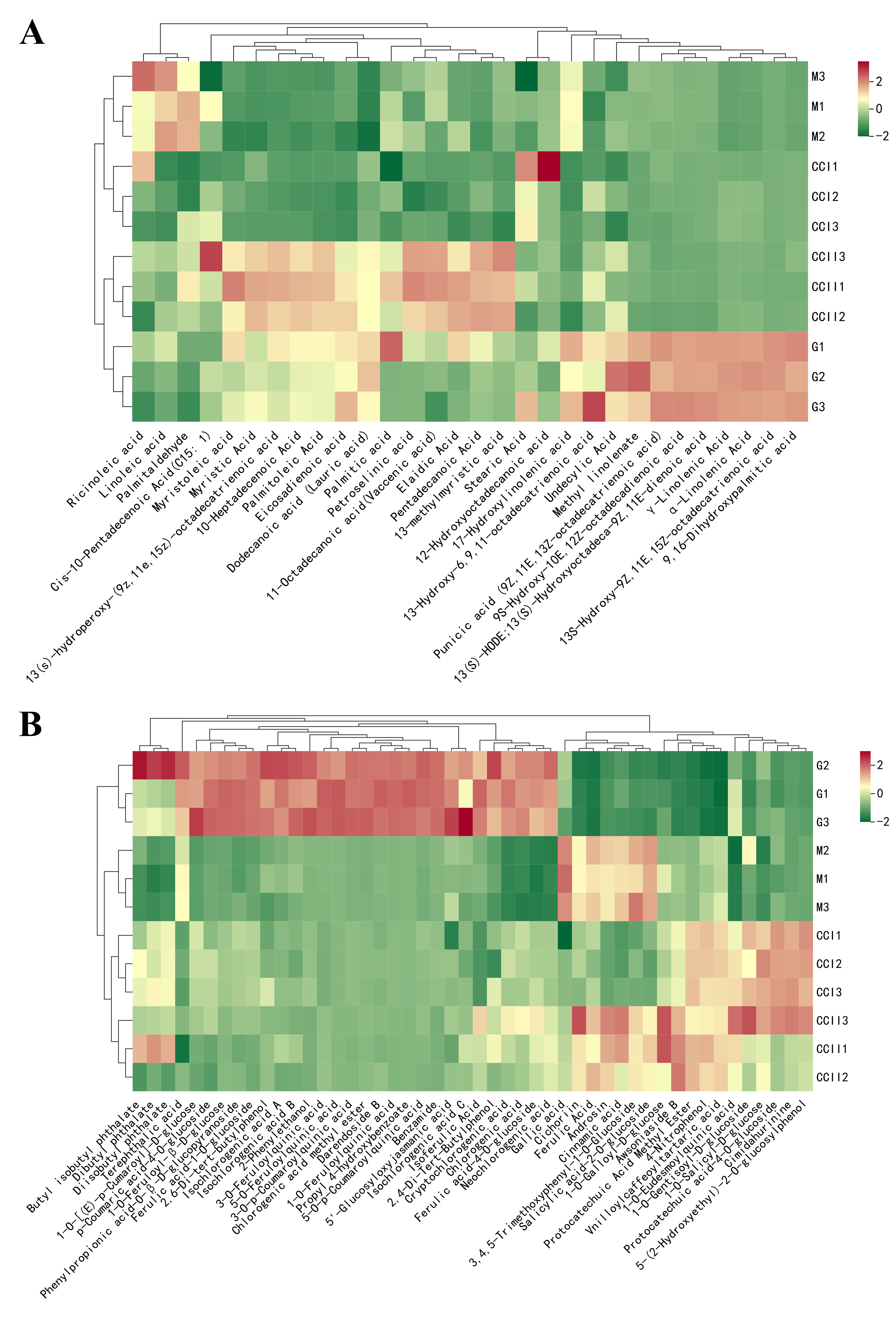

Supplement: Supplementary file 1 [file molecules-27-03870-s001.zip › Figure S3.jpg]

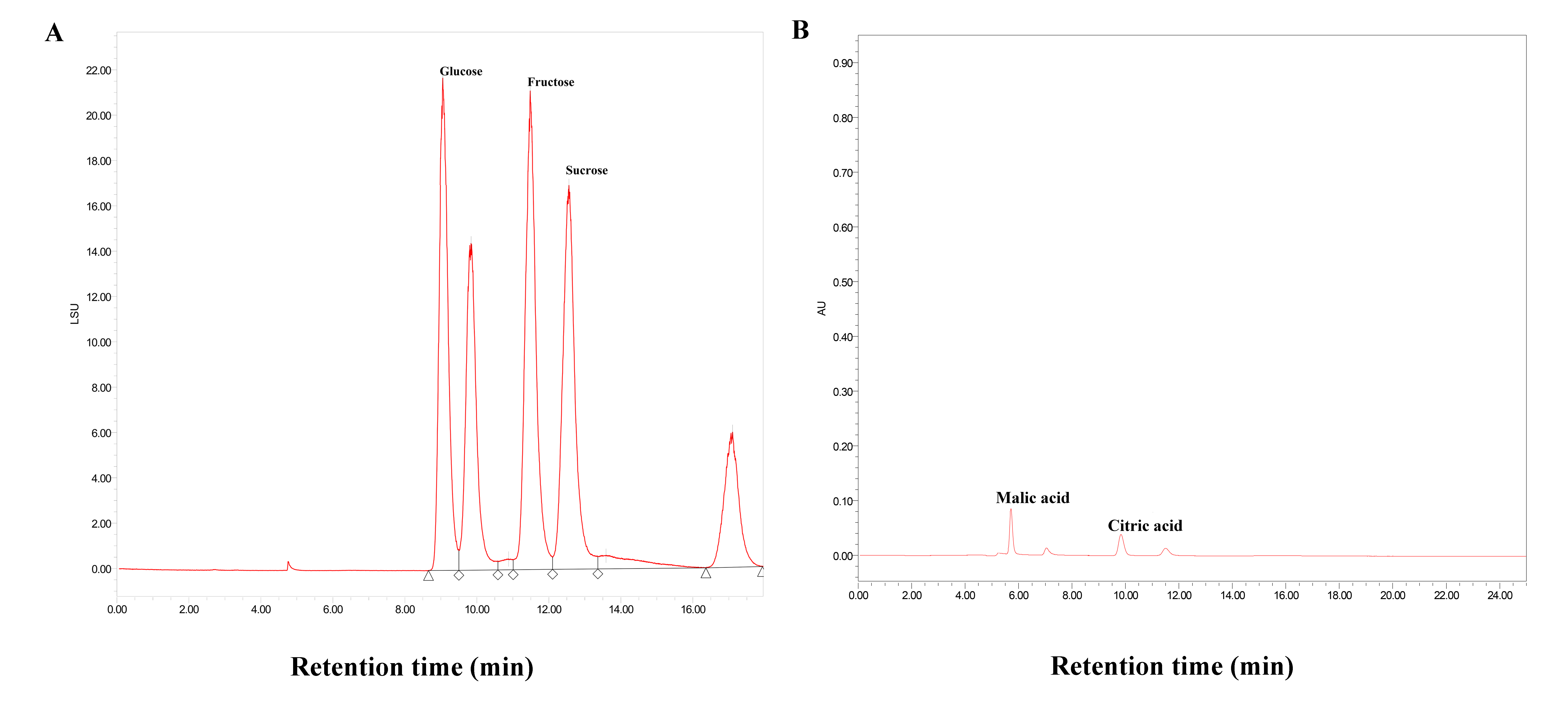

Supplement: Supplementary file 1 [file molecules-27-03870-s001.zip › Figure S4.tif]
